# Recent daily life burdens associated with neovascular age-related macular degeneration involve difficulties in use of electronic devices

**DOI:** 10.1038/s41598-024-65089-y

**Published:** 2024-06-20

**Authors:** Yoko Ozawa, Keisuke Yoshihara, Marwa Mezghani, Paulina Pierzchała, Mateusz Nikodem, Sylvaine Barbier, Mariko Nomoto, Yasuko Aitoku

**Affiliations:** 1 Department of Clinical Regenerative Medicine, Fujita Medical Innovation Center Tokyo, 1-1-4, Hanedakuko, Ota-ku, Haneda Innovation City Zone A, Tokyo, 144-0041 Japan; 2 Eye Center, Haneda Clinic, Fujita Health University, Tokyo, Japan; 3 Department of Ophthalmology, Keio University School of Medicine, Tokyo, Japan; 4 Bayer Yakuhin Ltd., Tokyo, Japan; 5 Putnam Associates, Tunis, Tunisia; 6 Putnam Associates, Cracow, Poland; 7 Putnam Associates, Lyon, France; 8 Putnam Associates, Tokyo, Japan

**Keywords:** Age-related macular degeneration, Patient survey, Questionnaire, Burden of disease, Network estimation, Electronic device, Diseases, Health care, Medical research

## Abstract

Neovascular age-related macular degeneration (nAMD) is a prevalent cause of permanent vision loss and blindness in the elderly worldwide, with a significant impact on patients’ daily lives. However, burdens related to nAMD from the patients’ perspective have not been well documented. Here we developed a new questionnaire after eliciting nAMD patients’ daily challenges followed by a pilot survey. Seven daily life burden domains were identified, and a quantitative survey was conducted using the questionnaire in the real-world clinic. Of the total 153 participants (mean age, 76.3 ± 8.3 years), 67 (43.8%) had bilateral nAMD, and 79 (52.7%) were classified into severe nAMD according to the best-corrected visual acuity with cut-off value of 0.52 in logMAR. Patients with bilateral and severe nAMD had significantly higher burden scores across all domains. Network models for the bilateral and severe disease subgroups identified the interactions between “activity of daily living” and “hand–eye coordination” and between “use of electronic devices” and “face recognition” domains, which were considered to be important burdens for the patients. These results can advance ophthalmologists’ understanding of the impact of nAMD on patients’ daily lives and the importance of active and continuing treatment for patients with nAMD.

## Introduction

Age-related macular degeneration (AMD) is a chronic and progressive disease of the retina, and is a leading cause of vision loss worldwide, affecting approximately 170 million individuals^1^. The prevalence of neovascular AMD (nAMD) in the Asian population aged 40–79 years is 0.56% and that in Caucasian populations is 0.59%, according to the previous meta-analysis of a population-based study in 2010^2^. The prevalence increased sharply by approximately 3 times from 2005 to 2013, according to the previous report based on the health insurance claims database in Japan^3^. Moreover, the risk for developing bilateral nAMD for individuals with unilateral nAMD within a 5 year period is approximately 19% to 28%^4^.

The treatment options available for nAMD include anti-vascular endothelial growth factor (anti-VEGF) therapy, photodynamic therapy, and laser therapy^5^. Anti-VEGF therapy was approved by the US Food and Drug Administration (FDA) in 2006 for the treatment of nAMD and is now widely recognized worldwide, as well as in Japan, as a standard of care for nAMD^6^. It is advantageous for final visual prognosis to initiate treatment for patients before they have experienced a significant loss in visual acuity^7–10^. However, it is also true that treatment discontinuation may negatively impact the prognosis^11–13^; the reported treatment retention rate decreased to 63% after a 3.5 year follow-up period^14^.

The nAMD-related quality of life (QOL) has been estimated by questionnaire health surveys which are developed for general health conditions^15,16^. The QOL and work probability were also reportedly reduced by 33.6% in the nAMD respondents compared with 16.2% in the control non-nAMD respondents^3^ using the Short Form (SF)-12v2 or SF-36v2 questionnaire health survey developed in 1996. Vision-related health components, such as reading, mobility, and emotions, were significantly impaired even when adjusting for visual acuity in Asian nAMD patients^17^ using the Impact of Vision Impairment questionnaire developed in 2002^18^. In addition, impairment of QOL in nAMD and improvement corresponding to treatment effects were reported previously^10^, using National Eye Institute 25-item Visual Function Questionnaire (NEI-VFQ-25) developed in 2001^19^. However, no previous questionnaires have been developed to quantify the vision-related burdens after the introduction of electronic technologies used on a daily basis. In addition, no previous studies have investigated the connections between nAMD daily life burden domains and patients’ background information.

In this study, we developed an original questionnaire, also incorporating daily burdens with recent lifestyles, and evaluated them using network analysis to show the association of the burden with patient backgrounds. The study will help clinicians and healthcare professionals, as well as patients, to understand the multi-perspective daily life burdens for nAMD. The newly developed questionnaire in this study will be of value to analyze how to improve clinical outcomes and QOL for patients in future studies.

## Results

Of the total 153 participants (mean age, 76.3 ± 8.3 years), 84 (54.9%) were male and 112 (73.2%) resided in the urban areas. Of them, 67 patients (43.8%) had bilateral nAMD, and 79 patients (52.7%) were classified into severe nAMD, with a best-corrected visual acuity (BCVA) 0.52 in logMAR (0.3 in decimal score of Landolt C chart) or worse in the affected eye of the patients with unilateral nAMD, and in the worse eye of the patients with bilateral nAMD. The data on BCVA were missing for 3 patients; these patients were excluded from the analyses according to the severity. There were 12 patients who had never had treatment for nAMD. The average duration of treatment for the 128 patients who clearly answered the questions related to nAMD treatment was 4 ± 3.6 years. The patients’ characteristics are presented in Table 1 for overall population and in Supplementary Table S1a,b for subgroup populations divided by laterality and severity, respectively.

**Table 1:** Patients’ background information for the overall population (N = 153).

Characteristics		n	All patients
Age	Mean (SD)	153	76.3 (8.3)
	Range (min; max)		(50.0; 93.0)
	Median (Q1; Q3)		77.0 (71.0; 83.0)
Sex	Female	153	69 (45.1%)
	Male		84 (54.9%)
Type of residence	Rural	153	41 (26.8%)
	Urban		112 (73.2%)
Employment status	Difficult to work	153	6 (3.9%)
	Housemaker		34 (22.2%)
	Others		13 (8.5%)
	Part-time worker		12 (7.8%)
	Permanent/full-time employee		10 (6.5%)
	Self-employed/employer		13 (8.5%)
	Unemployed or retiring age		65 (42.5%)
Disability welfare services	No	153	145 (94.8%)
	Yes		8 (5.2%)
Laterality	Unilateral	153	86 (56.2%)
	Bilateral		67 (43.8%)
BCVA (logMAR)*	Mean (SD)	150	0.63 (0.55)
	Range (min; max)		(− 0.08; no light perception)
	Median (Q1; Q3)		0.52 (0.17; 1.00)
Severity level**	Non-severe	150	71 (47.3%)
	Severe		79 (52.7%)
nAMD treatment	Yes	153	141 (92.2%)
	No		12 (7.8%)
If the above is yes, type of treatment	Pharmacotherapy (including anti-VEGF agents)	141	141 (100.0%)
	Photodynamic therapy		11 (7.8%)
	Other		3 (2.1%)
Treatment duration in years for nAMD	Mean (SD)	128	4.0 (3.6)
	Range (min; max)		(0.0; 18.0)
	Median (Q1; Q3)		3.0 (1.0; 6.0)

*BCVA* best-corrected visual acuity, *Min* minimum, *Max* maximum, *nAMD* neovascular age-related macular degeneration, *Q* quartile, *SD* standard deviation, *VEGF* vascular endothelial growth factor.

*It was asked decimal visual acuity in the questionnaire and converted into logMAR.

**Severe group, eyes with a BCVA 0.52 in logMAR (0.3 in decimal score of Landolt C chart) or worse in the affected eye of the patients with unilateral nAMD, and in the worse eye of the patients with bilateral nAMD.

### nAMD daily life burden score

**Figure 1:**
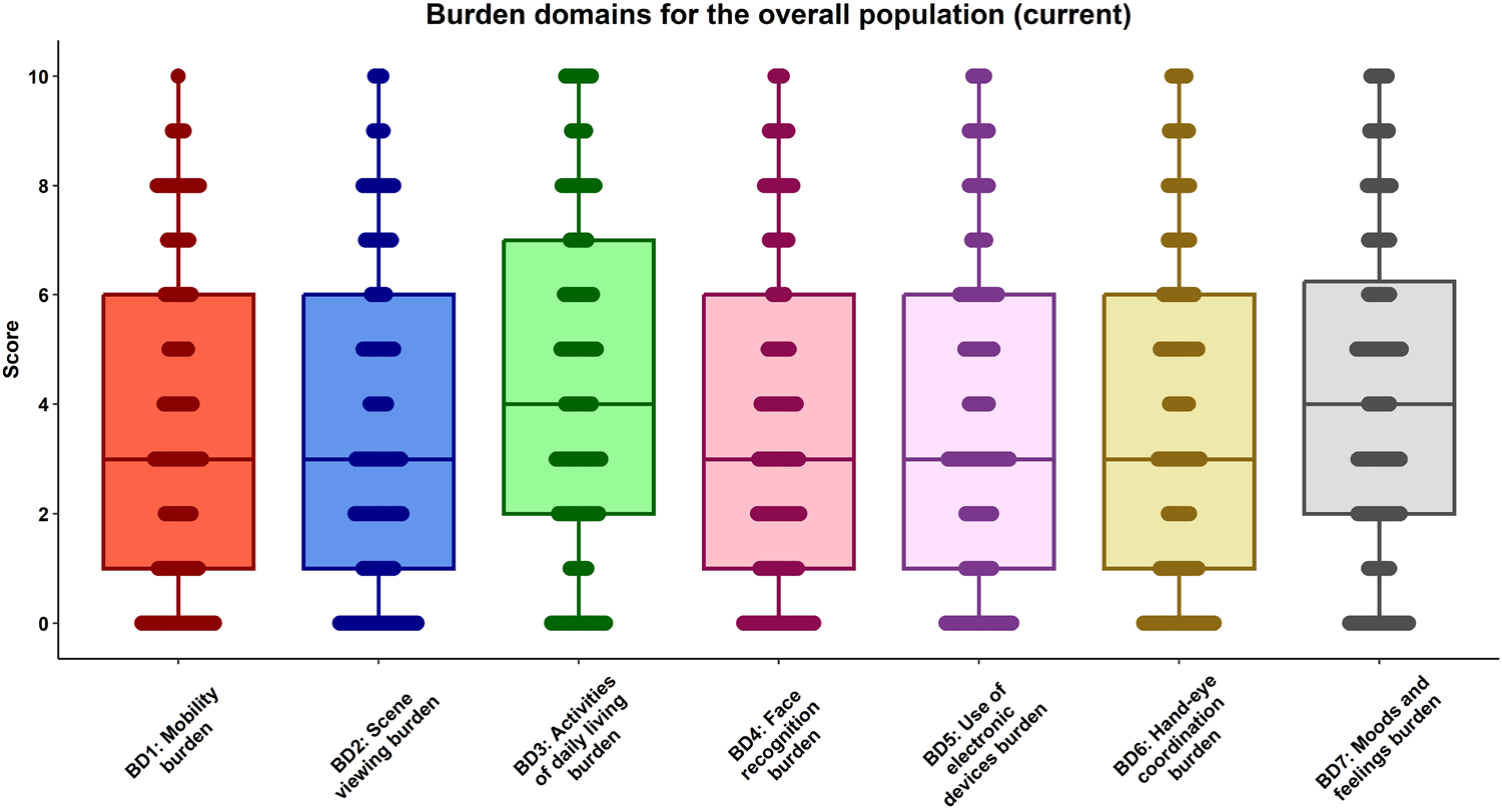
nAMD daily life burden domain scores for the overall population. The Box plots show the 5-number summary of a dataset: the minimum score (the end of the bottom whisker), first (lower) quartile (the lower line of the box), median (the line that divides the box into 2 parts), third (upper), quartile (the upper line of the box), and the maximum score (the end of the top whisker). The score 0 represents that the patients felt no inconvenience in performing the activity, and the score 10 represents that the patients felt that the activity was a severe burden and was hard to perform.

The main results show that the interquartile range was 1–6 in majority of nAMD daily life burden domains except for “activities of daily living”, which had a range of 2–7, and “moods and feelings”, which had a relatively narrow interval (2–6.25) as depicted in Fig. 1. Note that the score 0 represents that the patients felt no inconvenience and were able to perform the activity, and the score 10 represents the most severe burden and that the patients felt inconvenience and were hard to perform the activity. The detailed numerical results, such as, the mean with standard deviation and median with interquartile range, were presented in Supplementary Table S2.

### nAMD daily life burden score by subgroups

To evaluate the differences in nAMD daily life burden scores according to disease progression, the scores were compared between the bilateral and unilateral subgroups and between the severe and non-severe subgroups. In the comparison between bilateral and unilateral subgroups, the results showed that the daily life burden scores were significantly higher in all 7 domains in the bilateral subgroup (Table 2, Supplementary Fig. S1a–g). Furthermore, in the comparison between severe and non-severe subgroups, the severe subgroup presented significantly higher scores in all 7 domains (Table 2, Supplementary Fig. S1h–n).

**Table 2:** Burden scores according to the subgroups.

Domain	nAMD laterality			nAMD severity		
	Unilateral	Bilateral	P*	Severe	Non-severe	P*
Mobility burden (n)	86	67	< 0.001	79	71	< 0.001
Mean (SD)	2.8 (2.7)	4.7 (3.0)		4.8 (3.0)	2.4 (2.5)	
Range (min; max)	(0; 9)	(0; 10)		(0; 10)	(0; 9)	
Median (Q1; Q3)	2 (0; 4.7)	5 (2; 7.5)		5 (2; 8)	2 (0; 4)	
Scene viewing burden (n)	86	67	< 0.001	79	71	< 0.001
Mean (SD)	2.7 (2.6)	4.6 (3.2)		4.7 (3.2)	2.2 (2.3)	
Range (min; max)	(0; 10)	(0; 10)		(0; 10)	(0; 8)	
Median (Q1; Q3)	2 (0; 4)	5 (2; 7)		5 (2; 8)	2 (0; 3)	
Activities of daily living burden (n)	86	67	< 0.001	79	71	< 0.001
Mean (SD)	3.2 (2.7)	5.8 (3.1)		5.4 (3.0)	3.3 (3.0)	
Range (min; max)	(0; 10)	(0; 10)		(0; 10)	(0; 10)	
Median (Q1; Q3)	3 (1; 5)	6 (3.5; 8)		6 (3; 8)	3 (0.5; 5)	
Face recognition burden (n)	85	67	< 0.001	79	70	< 0.001
Mean (SD)	2.6 (2.6)	4.8 (3.1)		4.5 (3.3)	2.6 (2.4)	
Range (min; max)	(0; 10)	(0; 10)		(0; 10)	(0; 10)	
Median (Q1; Q3)	2 (0; 4)	5 (2.5; 8)		4 (2; 8)	2 (0.2; 4)	
Use of electronic devices burden (n)	84	66	< 0.001	78	69	< 0.001
Mean (SD)	2.8 (2.3)	5.3 (3.3)		4.9 (3.2)	2.8 (2.5)	
Range (min; max)	(0; 10)	(0; 10)		(0; 10)	(0; 10)	
Median (Q1; Q3)	3 (1; 4.2)	6 (3; 8)		5 (2.2; 8)	3 (0; 5)	
Hand–eye coordination burden (n)	86	67	< 0.001	79	71	< 0.001
Mean (SD)	2.9 (2.7)	5.3 (3.2)		5.1 (3.2)	2.7 (2.6)	
Range (min; max)	(0; 10)	(0; 10)		(0; 10)	(0; 10)	
Median (Q1; Q3)	3 (0; 5)	6 (3; 8)		5 (3; 8)	2 (0; 5)	
Moods and feelings burden (n)	85	67	< 0.001	78	71	< 0.001
Mean (SD)	3.2 (2.8)	5.2 (3.1)		5.1 (3.2)	3.0 (2.6)	
Range (min; max)	(0; 10)	(0; 10)		(0; 10)	(0; 10)	
Median (Q1; Q3)	3 (1; 5)	5 (3; 8)		5 (3; 8)	2 (1; 5)	

Burden scores were from 0 (with no inconvenience) to 10 (with inconvenience and/or unable to do).

*Min* minimum, *Max* maximum, *nAMD* neovascular age-related macular degeneration, *Q* quartile, *SD* standard deviation.

*P following unpaired Mann–Whitney–Wilcoxon test.

### Network analysis of nAMD daily life burdens and patients’ background information

**Figure 2:**
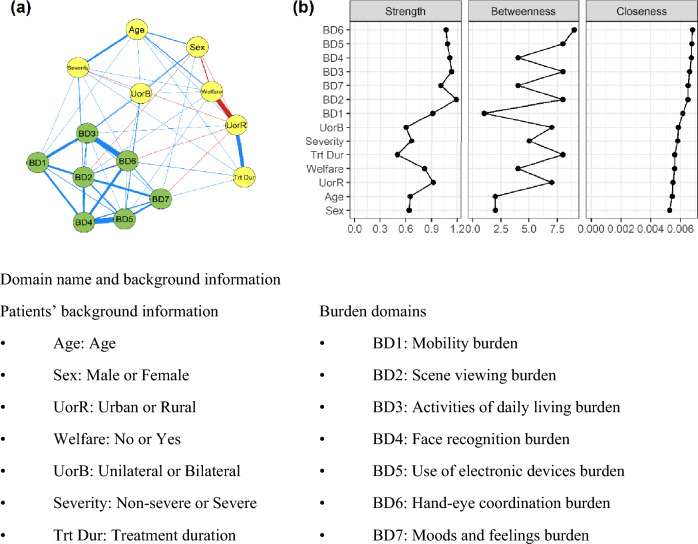
Network analysis of nAMD daily life burdens and background information in overall patients. (**a**) Network diagram. Two nodes are connected if the covariance between these 2 nodes cannot be explained by other nodes in the network. The thickness of the line represents the edge-weight value, which is the coefficient of the proportional partial correlation. The positive correlations are shown in blue and the negative in red. (**b**) Centrality indices for the network diagram for overall population.

The network diagram for the overall population revealed that the relatively high interaction between the patients’ background information and the daily life burden was observed between having bilateral nAMD and burdens regarding “use of electronic devices” (BD5) (Value of edges weight: 0.123) and “activities of daily living” (BD3) (Value of edges weight: 0.120) and between having severe nAMD and “scene viewing” (BD2) (Value of edges weight: 0.117). Values of edges weights for Fig. 2a were shown in Supplementary Table S3. Strong interactions were observed among multiple nAMD daily life burden domains (BD), particularly between “use of electronic devices” (BD5) and “face recognition” (BD4) (Value of edges weight: 0.344) and between “activities of daily living” (BD3) and “hand–eye coordination” (BD6) (Value of edges weight: 0.337) (Fig. 2a). On the other hand, the nodes representing patients’ background information showed only weak interaction with each other, except for the interactions between urban residence and the use of welfare services, implying that the welfare service users were more likely to be the citizens of urban areas, and between older age and severity.

The analysis of centrality indices in the disease network revealed that the BD2, BD3, BD5, and BD6 domains were the strong nAMD daily life burden domains in the overall network model, with sex being the least important factor (Fig. 2b).

### Network analyses in unilateral and bilateral subgroups

**Figure 3:**
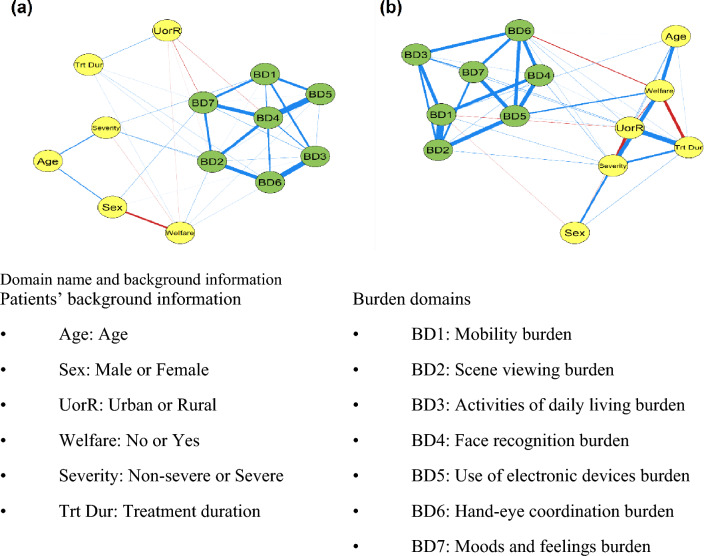
Network analysis of nAMD daily life burdens and background information in unilateral nAMD subgroup (**a**), and the bilateral nAMD subgroup (**b**). Two nodes are connected if the covariance between these 2 nodes cannot be explained by other nodes in the network. The thickness of the line represents the value of edges weight, which is the coefficient of the proportional partial correlation. (The positive value is shown in blue, and the negative in red).

The network model subgroup analyses were further performed after dividing the patients into unilateral (Fig. 3a) and bilateral (Fig. 3b) subgroups. The correlations between burden domain nodes were generally strong in the bilateral subgroup, linking to multiple patient backgrounds. In the network diagram generated for the bilateral subgroup, the edges connecting the burden domains to the nodes representing patients’ background information had high absolute weights in the majority of cases, while in the network diagram for the unilateral patient subgroup, correlations were weak among the burden domains and background information. Values of edges weights for Fig. 3b were shown in Supplementary Table S4.

### Network analyses in non-severe and severe subgroups

**Figure 4:**
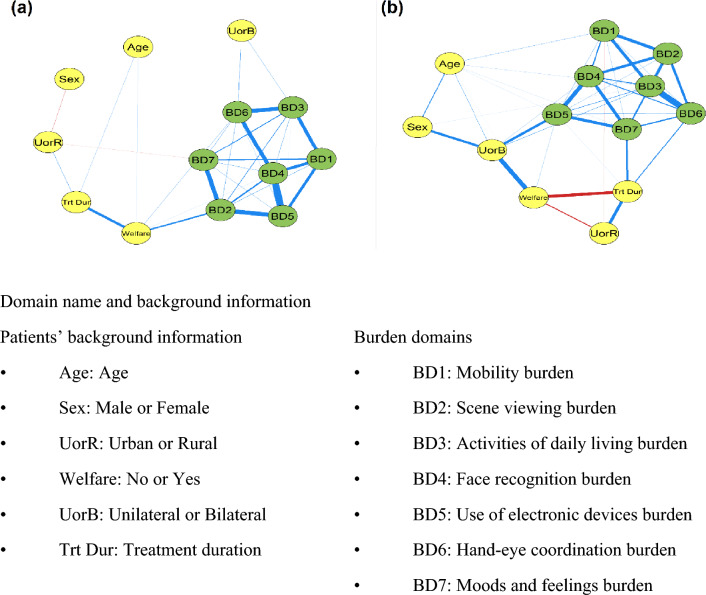
Network analysis of nAMD daily life burdens and background information in the (**a**) non-severe subgroup and (**b**) severe subgroup. Two nodes are connected if the covariance between these 2 nodes cannot be explained by other nodes in the network. The thickness of the line represents the value of edges weight, which is the coefficient of the proportional partial correlation. (The positive value is shown in blue, and the negative in red).

Network analyses were separately performed for the nAMD non-severe (Fig. 4a) and severe (Fig. 4b) subgroups. In the network diagram generated for the severe subgroup, the edge-weight values connecting the burden domains with each other, and burden domain to the nodes representing patients’ background information were high in the majority of cases, while it was not in those for the non-severe patient subgroup (Fig. 4a,b). In the network estimated for the severe subgroup of patients, both the demand for social welfare services and BD5 interacted with nAMD laterality being bilateral (Value of edges weight: 0.245 and 0.191 respectably). Values of edges weights for Fig. 4b were shown in Supplementary Table S5.

### Evaluation of burdens using validated questionnaires

The scores of other validated patient-reported outcomes (PROs) and QOL summary for the overall population are presented in Table 3. The average NEI-VFQ-25 score was 67.4 (SD = 19.2), and over half of the patients (56%) were classified as having “Non/Minimal” severity in the PHQ-9 score. The mean EQ-5D-5L utility value was 0.85 (SD = 0.2) for the overall population. Detailed results on the validated QOL summary scores by nAMD laterality and severity subgroups are presented in Supplementary Table S6a,b, respectively, showing statistical differences between the subgroups where the scores were higher in the bilateral and severe nAMD subgroups.

**Table 3:** Validated QOL summary scores for the overall population (N = 153).

QOL score		n	All patients
NEI-VFQ-25 score	Mean (SD)	153	67.4 (19.2)
	Range (min; max)		(19.1; 97.9)
	Median (Q1; Q3)		71.3 (54.1; 82.7)
PHQ-9 summary score	0–4 none/minimal	150	84 (56.0%)
	5–9 mild		44 (29.3%)
	10–14 moderate		14 (9.3%)
	15–19 moderately severe		7 (4.7%)
	20–27 severe		1 (0.7%)
EQ-5D-5L	Mean (SD)	149	0.8 (0.2)
	(Min; max)		(0.3; 1)
	Median (Q1; Q3)		0.9 (0.8; 1)

The score ranged from 0 to 100 (QOL: better) on the NEI-VFQ-25, from 0 to 27 (depression severity: severe) on the PHQ-9 summary, and from 0 (a state as bad as being dead) to 1 (full health) on the EQ-5D-5L questionnaires.

*EQ-5D-5L* EuroQOL 5-Dimension 5-Level Questionnaire, *Min* minimum, *Max* maximum, *nAMD* neovascular age-related macular degeneration, *NEI-VFQ-25* national eye institute 25-item visual function questionnaire, *PHQ-9* patient health questionnaire-9, *Q* quartile, *QOL* quality of life, *SD* standard deviation.

### NEI-VFQ-25 subcategories by subgroups

The detailed results for NEI-VFQ-25 lower domains for the overall population are presented in Supplementary Table S7a. The results of the NEI-VFQ-25 lower domains by nAMD laterality showed that scores for all subcategories were significantly lower in the bilateral subgroup except for the “general health” subdomain as shown in Supplementary Fig. S2.

The results of the NEI-VFQ-25 subcategories by subgroups showed that scores for all subcategories were significantly lower in the severe subgroup except for the “general health” and “driving” subdomains as shown in Supplementary Fig. S3. Numerical results for Supplementary Figs. S1 and S2 can be found in Supplementary Table S7b,c. The associations between the scores of the newly developed questionnaire in the current study and NEI-VFQ-25 were shown in Supplementary Table S8.

## Discussion

In this study, we developed a novel questionnaire and examined the burden associated with the QOL of patients with nAMD; in addition, we explored the relationship between the daily burden domains of nAMD and patients’ background information. The sample included participants with a diverse range of characteristics, comprising both rural and urban residents, patients with both bilateral and unilateral disease, and those with severe and non-severe cases of nAMD defined by BCVA. Analysis of the data revealed statistically significant differences in all daily burden domains of nAMD when comparing the bilateral and unilateral disease subgroups, and severe and non-severe nAMD subgroups.

The questionnaire was developed based on the one-on-one interviews with nAMD patients in the current study. The questions were concrete, reflecting the real-world nAMD patients’ complaints (details in the Supplementary information). We found that patients of relatively older age reported desire for use of electronic devices such as computers, cell phones, and bank ATMs by themselves, as reflected by modern lifestyles. Elder people may use online shopping if they feel hard to walk around by themselves, and they may carry cell phones so that they can contact in anytime including in case of emergency. Thus, it would be important for the elder people to use electronic devices.

In the new questionnaire, the interquartile burden scores ranged 1 or 2 to 6 or 7, and the answers varied according to the individuals. However, the scores were greater in the bilateral group compared with unilateral group, and severe group compared with non-severe group, which support the credibility of the questionnaire.

The results from the network show which burden domains affect patients greatly, as well as whether such domains interact strongly with each other through quantification of the magnitude of burden, which provides a path forward for clinicians to recognize the most important factors to improve patients’ QOL.

The network approach allowed evaluation of various data points and provided valuable insights by integrating physical, psychological, and demographic data, and it showed that the physical and psychological burden of nAMD has a significant impact on the daily lives of patients, especially for the bilateral and severe subpopulations.

The overall results of the study showed that in all of the networks, regardless of the subgroups, the most important links between the nodes representing different burden domains of nAMD were observed between “activities of daily living” (BD3) and “hand–eye coordination” burdens (BD6), and between “use of electronic devices” (BD5) and “face recognition” burdens (BD4). This suggests that the different burden domains interact strongly and suggested that they may have a significant impact on one another. Additionally, the strong links between the different domains suggest that interventions aimed at reducing one domain may have a positive impact on other domains as well.

In the network estimated for the bilateral subgroup as well as the severe subgroup, the “use of electronic device” burden (BD5) had relatively a high interaction with the “face recognition” burden (BD4) and “moods and feelings” burden (BD7). The associations were partly supported by the results of NEI-VFQ-25. Those who chose high score of “use of electronic device” burden (BD5) also expressed great difficulties in distance activity and a bad mental health in NEI-VFQ-25. We interpreted that paying attention to the electronic device (digital tool) use by patients could be a useful way of monitoring their daily burden. Furthermore, our findings suggest that activities of daily living, hand–eye coordination, and scene viewing burdens play a central role in the disease burden experienced by those patients and should be given particular attention in future studies and interventions aimed at reducing the overall disease burden.

With certain exceptions, the connections between the nodes representing patients’ background information, or the connections between patients’ background information and the burden domains, had negligible or no weight. The limited importance of patients’ sex in the network indicates that it may have a limited influence on the overall burden of the disease. However, the relationship between the urban residence and welfare services highlights the importance of considering patients’ background information in assessing and addressing the burden experienced by patients.

The NEI-VFQ scale is a well-established, recognized, and validated vision-targeted QOL scale. The results of the questionnaire developed in the current study from the same patients were consistent with the NEI-VFQ, indicating that our scale captures the QOL in patients with eye diseases in a similar way. However, the newly developed questionnaire in the current study included the burden related to the use of electronic devices, which was not taking into account in the NEI-VFQ. We observed that this burden had a strong interaction with the other burdens, which highlights the relevance of including this dimension to effectively capture modern lifestyle factors. Additionally, the general QOL scales, such as PHQ-9 and EQ-5D-5L, were not able to properly reflect the burden experienced by nAMD patients in their daily life. The proposed new scale is able to capture disease progression with greater sensitivity for each burden domain and is easily understood by patients since it reflects their current lifestyle.

This study has certain limitations that should be considered when interpreting the results. Firstly, the sample size in this study may not be sufficient to draw conclusions about the stability of the whole network of interactions between nAMD and patients’ QOL. Secondly, the study population may not be fully representative of the overall population of patients with nAMD in Japan. Specifically, the proportions of patients with bilateral and severe nAMD were kept intentionally higher in this study compared with the real-world prevalence. It is important to note that these results should be further confirmed through larger-scale studies^20^. Thirdly, although the study results suggested a relationship between nAMD and impaired patients’ QOL, it is possible that other factors besides nAMD might have also affected the QOL in these patients. Lastly, patients were recruited through eye clinics with experience in nAMD treatment, and such patients may have had milder disease due to ongoing treatment, which may not allow reflection of the fundamental burdens of nAMD. However, the scores in the bilateral and severe groups were meaningful even after treatment.

In conclusion, the newly developed questionnaire captured burdens related to daily life in patients with visual impairment due to nAMD. Not only seeing and moving, but use of electronic devices was also an important activity for the patients. The network estimation model provides an insight into the complex nature of the disease burden and its relationship with patients’ background information. Moreover, the impact of the burdens, and relationships between the burdens, and between burden and background information, differed according to the laterality and severity of nAMD. Understanding the burdens associated with nAMD is important for guiding clinicians’ treatment and management of nAMD, for example, enthusiasm for early initiation and long-term continuation of nAMD treatment, as well as for patients, as it may motivate them to adhere to treatment and ultimately lead to improved clinical outcomes and QOL for patients.

## Materials and methods

The study was approved by the central institutional review board at the Research Institute of Healthcare Data Science (RIHDS). This study was conducted in accordance with the ethical principles outlined in the Declaration of Helsinki and followed the guidelines of good clinical practices and the applicable laws and regulations of Japan. Informed consent was obtained from all subjects involved in the study.

### Study design

This study was composed of 3 steps. Step 1, i.e., “Concept Elicitation” aimed to assess patients’ daily life burdens by conducting one-on-one interviews with 5 patients with AMD at the Ishida Eye Clinic (Saitama, Japan). The interview guide was developed based on the AMD burden systematic review published by Taylor et al.^21^. Seven daily life burden domains of nAMD were identified, such as mobility, scene viewing, activities of daily living, face recognition, use of electronic devices, hand–eye coordination, and moods and feelings. The data gathered in this step were then used to conceptualize a draft questionnaire covering the 7 burden domains that would be tested in the subsequent step.

Step 2, i.e., “Pilot Survey & Cognitive Interview” aimed to test the questionnaire created in Step 1. In this step, 5 patients with AMD were asked to provide feedback on the questionnaire usability, language accuracy, and other aspects that could be improved prior to finalization, and this was also performed at the Ishida Eye Clinic.

Lastly, Step 3, i.e., “Quantitative Patient Survey” was conducted. The survey utilized demographic and medical history questions. For the finalized questionnaire, which we developed, the patients were asked to choose their answers from a scale of 0 (no inconvenience) to 10 (the most severe burden) for each question. Several other published questionnaires, such as the National Eye Institute 25-Item Visual Function Questionnaire (NEI-VFQ-25), a self-reported measure of vision-related status^22^; the Patient Health Questionnaire-9 (PHQ-9), a patient-reported questionnaire for depression^23^; and the EuroQol-5 Dimension 5-Level Questionnaire (EQ-5D-5L), a patient-reported questionnaire for general health^24^ were also used after obtaining permission from the copyright holder prior to the initiation of the study. If patients did not answer the question or answered, “do not know”, those responses were treated as missing data.

The quantitative patient survey was conducted with a target sample size of 150 Japanese patients with nAMD, who were recruited from 13 geographically dispersed eye clinics in Japan. Only the board-certified ophthalmologists with a minimum of 5 years of clinical experience and seeing at least 5 patients with nAMD per month were included in the study. In addition, geographical location of clinics was considered for site selection to be balanced between urban and rural areas based on the Organization for Economic Cooperation and Development (OECD) typology^25^. Participants who were more than 50 years old were included in this study only if they had clinically diagnosed nAMD, were able to understand and answer the questionnaire by themselves, and agreed to sign the written informed consent. Participants who had a hearing impairment were excluded from the study. The minimum sample size of 150 was estimated based on a simulation to ensure sufficient precision of the correlation coefficients in the network analyses. Participants were compensated for their time spent on the study in accordance with the ethical board. BCVA was measured to classify the patients into severe nAMD in the same eye clinic where they were recruited.

### Statistical analysis

Descriptive statistics were performed to summarize patients’ background information, nAMD daily life burden domains, and validated QOL summary scores (NEI-VFQ-25 score and lower domains, PHQ-9, and EQ-5D-5L utility), excluding the missing and invalid observations that occurred because of the data collection being survey based. The answers to the present burden at the time of the study were analyzed.

Patients were divided into “unilateral” and “bilateral” subgroups according to the nAMD status of affected eyes, and into “severe” and “non-severe” subgroups based on BCVA and the differences in burden scores were assessed with Mann–Whitney U tests.

Multivariable disease network models were constructed to investigate the relationship between nAMD daily life burden domains and patients’ background information in order to understand the complexity of nAMD disease in the target population^20,26^. The network consisted of nodes representing the nAMD daily life burden domains and patients’ background information and edges representing the partial correlations between the nodes. The graphical least absolute shrinkage and selection operator (LASSO) regularization technique was used to shrink edges of small absolute weight and constrain them to zero. The following steps were taken to construct the optimal network: (i) estimate partial correlation network, (ii) regularize the network with the LASSO penalization multiple times using different tuning parameters (λ varying from 0 to 1), and (iii) select the optimal network among multiple networks by minimizing the Extended Bayesian Information Criterion (EBIC). The most important nodes were placed in the center of the network based on the 3 theoretical centrality measures: strength, closeness, and betweenness centrality indices. The node strength was calculated by summing the “strengths” of all edges connected to the node, where the edge strength is an absolute value of edge weight. The node “betweenness” was defined by the number of shortest paths between 2 nodes that go through the node. Finally, the node “closeness” was the inverse of the sum of all the shortest paths between the node and all other nodes in the network. For all the centrality measures, the interpretation was the same: the higher the value, the more central the node was.

The binary variables were decoded as follows: For higher values with higher severity or burden—unilateral = 1, bilateral = 2; non-severe = 1, severe = 2; welfare service usage “no” = 1, welfare service usage “yes” = 2. For the nodes that could not be classified by severity level, the code used was as follows: male = 1, female = 2; urban = 1, rural = 2.

Additionally, networks for the subgroups “unilateral”, “bilateral”, “severe”, and “non-severe” were generated.

For the analyses of the associations between the results of the newly developed questionnaire in the current study and NEI-VFQ-25, Pearson’s correlation coefficient was used.

The statistical analysis was conducted using R: A Language and Environment for Statistical Computing, version 4.1.2^27^, with the bootnet package applied for the network analysis^26,28^. P values < 0.05 were considered statistically significant.

### Supplementary Information

Supplementary Information.

## Data Availability

In this study, it was decided that patient-reported data would not be made public due to its confidentiality. The data generated and analyzed during the study are not publicly available due to the patent of Bayer but might be available from the corresponding author on reasonable request.
