# Supplementary material for: Recent daily life burdens associated with neovascular age-related macular degeneration involve difficulties in use of electronic devices
Source: Sci Rep. 2024 Jun 20;14:14181. doi: 10.1038/s41598-024-65089-y (PMC11187225; doi:10.1038/s41598-024-65089-y)
Supplement: Supplementary file 1 — Supplementary Information. [file 41598_2024_65089_MOESM1_ESM.pdf]

Recent daily life burdens associated with neovascular age-related macular degeneration involve difficulties in use of electronic devices

Yoko Ozawa[1,2,3]*, Keisuke Yoshihara[4], Marwa Mezghani[5], Paulina Pierzchała[6], Mateusz Nikodem[6], Sylvaine Barbier[7], Mariko Nomoto[8], Yasuko Aitoku[4]

1. Department of Clinical Regenerative Medicine, Fujita Medical Innovation Center TOKYO, Tokyo Japan
2. Eye Center, Fujita Health University, Haneda Clinic, Tokyo Japan;
3. Department of Ophthalmology, Keio University School of Medicine, Tokyo, Japan;
4. Bayer Yakuhin Ltd., Tokyo, Japan
5. Putnam Associates, Tunis, Tunisia
6. Putnam Associates, Cracow, Poland
7. Putnam Associates, Lyon, France
8. Putnam Associates, Tokyo, Japan

*Correspondence author
Yoko Ozawa M.D., Ph.D
Professor
Department of Clinical Regenerative Medicine
Fujita Medical Innovation Center Tokyo
Eye Center, Fujita Health University, Haneda Clinic
1-1-4, Hanedakuko, Ota-ku,
Haneda Innovation City Zone A,
Tokyo 144-0041
Tel; +81-3-5708-7830
ozawa@a5.keio.jp, yoko.ozawa@fujita-hu.ac.jp
ORCID: 0000-0003-4797-5705

Supplementary Table S1 (a). Patients' background information according to nAMD laterality.

**Characteristics**	**Unilateral**	**Bilateral**	**P***
Age (n)	86	67	**0.003**
Mean (SD)	74.6 (7.7)	78.4 (8.6)	
Range (Min; Max)	(52; 89)	(50; 93)	
Median (Q1; Q3)	74 (70; 80)	79 (74; 85)	
Sex (n)	86	67	0.084
Female	33 (38.4%)	36 (53.7%)	
Male	53 (61.6%)	31 (46.3%)	
Type of residence (n)	86	67	0.867
Rural	24 (27.9%)	17 (25.4%)	
Urban	62 (72.1%)	50 (74.6%)	
Employment status (n)	86	67	0.120
Difficult to work	2 (2.3%)	4 (6.0%)	
Housemaker	20 (23.3%)	14 (20.9%)	
Others	7 (8.1%)	6 (8.9%)	
Part-time worker	8 (9.3%)	4 (6.0%)	
Permanent / Full-time employee	9 (10.5%)	1 (1.5%)	
Self-employed / Employer	4 (4.6%)	9 (13.4%)	
Unemployed or retiring age	36 (41.9%)	29 (43.3%)	
Disability welfare services (n)	86	67	0.299
No	83 (96.5%)	62 (92.5%)	
Yes	3 (3.5%)	5 (7.5%)	
BCVA** (logMAR) (n)	83	67	**<0.001**
Mean (SD)	0.49 (0.47)	0.81 (0.92)	
Range (Min; Max)	(-0.08; no light perception)	(-0.08; no light perception)	
Median (Q1; Q3)	0.4 (0.15; 0.7)	0.7 (0.3; 1.22)	
Severity level*** (n)	83	67	**0.018**
Non-severe	47 (56.6%)	24 (35.8%)	
Severe	36 (43.4%)	43 (64.2%)	
Treatment (n)	86	67	0.882
Yes	80 (94.2%)	61 (95.5%)	
No	6 (5.8%)	6 (4.5%)	
If the above is yes, type of treatment (n)	80	61	
Pharmacotherapy (including anti-VEGF agents)	80 (100.0%)	61 (100.0%)	1
Photodynamic therapy	4 (5.0%)	7 (11.5%)	0.208
Other	1 (1.2%)	2 (3.3%)	0.578
Treatment duration in years for nAMD (n)	73	55	0.137
Mean (SD)	3.6 (3.4)	4.6 (3.7)	
Range (Min; Max)	(0.1; 12)	(0; 18)	
Median (Q1; Q3)	2 (1; 5)	4 (2; 7)	

Abbreviations: BCVA, best-corrected visual acuity; Min, minimum; Max, maximum; nAMD, neovascular age-related macular degeneration; Q, quartile; SD, standard deviation; VEGF, vascular endothelial growth factor.
*P following Chi-square or Fisher's exact test (if more than 20% of subcategories have frequencies lower than 5) in categorical variables and unpaired Mann-Whitney-Wilcoxon test in continuous variables.
**It was asked decimal acuity in the questionnaire and calculated into logMAR.
***Severe group, eyes with a BCVA 0.52 in logMAR (0.3 in decimal score of Landolt C chart) or worse in the affected eye of the patients with unilateral nAMD, and in the worse eye of the patients with bilateral nAMD.

Supplementary Table S1 (b). Patients' background information according to nAMD severity.

**Characteristics**	**Severe**	**Non-severe**	**P***
Age (n)	79	71	**0.005**
Mean (SD)	78.2 (7.5)	73.9 (8.6)	
Range (Min; Max)	(59; 93)	(50; 89)	
Median (Q1; Q3)	78 (73.5; 83.5)	74 (69.5; 80)	
Sex (n)	79	71	0.377
Female	39 (49.4%)	29 (40.8%)	
Male	40 (50.6%)	42 (59.2%)	
Type of residence (n)	79	71	0.256
Rural	18 (22.8%)	23 (32.4%)	
Urban	61 (77.2%)	48 (67.6%)	
Employment status (n)	79	71	**0.033**
Difficult to work	6 (7.6%)	0 (0%)	
Housemaker	16 (20.2%)	17 (23.9%)	
Others	4 (5.1%)	9 (12.7%)	
Part-time worker	6 (7.6%)	6 (8.4%)	
Permanent / Full-time employee	3 (3.8%)	7 (9.9%)	
Self-employed / Employer	5 (6.3%)	8 (11.3%)	
Unemployed or retiring age	39 (49.4%)	24 (33.8%)	
Disability welfare services (n)	79	71	0.281
No	73 (92.4%)	69 (97.2%)	
Yes	6 (7.6%)	2 (2.8%)	
Laterality (n)	79	71	**0.018**
Unilateral	36 (45.6%)	47 (66.2%)	
Bilateral	43 (54.4%)	24 (33.8%)	
BVCA** (n)	79	71	**<0.001**
Mean (SD)	1.05 (0.44)	0.17 (0.15)	
Range (Min; Max)	(0.52; no light perception)	(-0.08; 0.4)	
Median (Q1; Q3)	1 (0.7; 1.40)	0.15 (0.05; 0.3)	
Treatment (n)	79	71	0.621
Yes	74 (94.9%)	64 (94.4%)	
No	5 (5.1%)	7 (5.6%)	
If the above is yes, type of treatment (n)	74	64	
Pharmacotherapy (including anti-VEGF agents)	74 (100.0%)	64 (100.0%)	1
Photodynamic therapy	10 (13.5%)	1 (1.6%)	**0.01**
Other	2 (2.7%)	1 (1.6%)	1
Treatment duration in years for nAMD (n)	67	59	0.213
Mean (SD)	4.5 (3.9)	3.6 (3.1)	
Range (Min; Max)	(0.0; 18)	(0.1; 12)	
Median (Q1; Q3)	4 (1; 7)	3 (1; 5)	

Abbreviations: BCVA, best-corrected visual acuity; Min, minimum; Max, maximum; nAMD, neovascular age-related macular degeneration; Q, quartile; SD, standard deviation; VEGF, vascular endothelial growth factor.
*P following Chi-square or Fisher's exact test (if more than 20% of subcategories have frequencies lower than 5) in categorical variables and unpaired Mann-Whitney-Wilcoxon test in continuous variables.
**Severe group, eyes with a BCVA 0.52 in logMAR (0.3 in decimal score of Landolt C chart) or worse in the affected eye of the patients with unilateral nAMD, and in the worse eye of the patients with bilateral nAMD.

Supplementary Table S2. Burden scores for the overall population.

Domain	All patients
Mobility burden (n)	153
Mean (SD)	3.7 (3.0)
Range (Min; Max)	(0; 10)
Median (Q1; Q3)	3 (1; 6)
Scene viewing burden (n)	153
Mean (SD)	3.5 (3.0)
Range (Min; Max)	(0; 10)
Median (Q1; Q3)	3 (1; 6)
Activities of daily living burden (n)	153
Mean (SD)	4.4 (3.2)
Range (Min; Max)	(0; 10)
Median (Q1; Q3)	4 (2; 7)
Face recognition burden (n)	152
Mean (SD)	3.6 (3.1)
Range (Min; Max)	(0; 10)
Median (Q1; Q3)	3 (1; 6)
Use of electronic devices burden (n)	150
Mean (SD)	3.9 (3.0)
Range (Min; Max)	(0; 10)
Median (Q1; Q3)	3 (1; 6)
Hand-eye coordination burden (n)	153
Mean (SD)	3.9 (3.1)
Range (Min; Max)	(0; 10)
Median (Q1; Q3)	3 (1; 6)
Moods and feelings burden (n)	152
Mean (SD)	4.1 (3.1)
Range (Min; Max)	(0; 10)
Median (Q1; Q3)	4 (2; 6.2)

Abbreviations: Min, minimum; Max, maximum; Q, quartile; SD, standard deviation.
Burden scores were from 0 (with no inconvenience) to 10 (with inconvenience and/or unable to do).

Supplementary Table S3. Edges weights based on the partial correlation coefficients for overall sample network (where the absolute value is >0.1).

Node 1	Node 2	Value
BD5: Use of electronic devices burden	BD4: Face recognition burden	0.344
BD3: Activities of daily living burden	BD6: Hand-eye coordination burden	0.337
UoR: Urban or Rural	Trt Dur: Treatment duration	0.256
BD1: Mobility burden	BD3: Activities of daily living burden	0.221
BD4: Face recognition burden	BD6: Hand-eye coordination burden	0.196
BD7: Moods and feelings burden	BD2: Scene viewing burden	0.192
BD1: Mobility burden	BD2: Scene viewing burden	0.184
BD2: Scene viewing burden	BD4: Face recognition burden	0.177
Age	Severity	0.175
BD1: Mobility burden	BD4: Face recognition burden	0.171
BD7: Moods and feelings burden	BD5: Use of electronic devices burden	0.170
BD2: Scene viewing burden	BD6: Hand-eye coordination burden	0.144
BD2: Scene viewing burden	BD5: Use of electronic devices burden	0.139
BD7: Moods and feelings burden	BD4: Face recognition burden	0.138
Age	Sex	0.132
Age	Welfare	0.130
UorB: Unilateral or Bilateral	BD5: Use of electronic devices burden	0.123
UorB: Unilateral or Bilateral	BD3: Activities of daily living burden	0.120
Sex	UorB: Unilateral or Bilateral	0.119
BD2: Scene viewing burden	BD3: Activities of daily living burden	0.119
BD7: Moods and feelings burden	BD6: Hand-eye coordination burden	0.118
Severity	BD2: Scene viewing burden	0.117
BD1: Mobility burden	BD5: Use of electronic devices burden	0.112
BD7: Moods and feelings burden	BD3: Activities of daily living burden	0.109
Trt Dur: Treatment duration	BD6: Hand-eye coordination burden	0.103
Sex	Welfare	-0.131
UoR: Urban or Rural	Welfare	-0.322

Supplementary Table S4. Edges weights based on the partial correlation coefficients for bilateral subgroup network (where the absolute value is >0.1).

Node 1	Node 2	Value
BD1: Mobility burden	BD2: Scene viewing burden	0.386
UorR: Urban or Rural	Trt Dur: Treatment duration	0.331
BD2: Scene viewing burden	BD5: Use of electronic devices burden	0.285
BD4: Face recognition burden	BD5: Use of electronic devices burden	0.262
Welfare	Severity	0.261
BD5: Use of electronic devices burden	BD7: Moods and feelings burden	0.259
BD3: Activities of daily living burden	BD6: Hand-eye coordination burden	0.249
BD1: Mobility burden	BD4: Face recognition burden	0.249
Age	Welfare	0.233
BD4: Face recognition burden	BD6: Hand-eye coordination burden	0.226
BD1: Mobility burden	BD3: Activities of daily living burden	0.225
BD5: Use of electronic devices burden	BD6: Hand-eye coordination burden	0.218
BD6: Hand-eye coordination burden	BD7: Moods and feelings burden	0.209
Severity	Trt Dur: Treatment duration	0.187
BD2: Scene viewing burden	BD7: Moods and feelings burden	0.174
Sex	Severity	0.173
BD2: Scene viewing burden	BD3: Activities of daily living burden	0.167
Welfare	BD5: Use of electronic devices burden	0.153
BD3: Activities of daily living burden	BD7: Moods and feelings burden	0.136
UorR: Urban or Rural	Welfare	-0.110
Welfare	BD6: Hand-eye coordination burden	-0.124
Welfare	Trt Dur: Treatment duration	-0.205
UorR: Urban or Rural	Severity	-0.211

Supplementary Table S5. Edges weights based on the partial correlation coefficients for severe subgroup network (where the absolute value is >0.1).

Node 1	Node 2	Value
BD3: Activities of daily living burden	BD6: Hand-eye coordination burden	0.324
Welfare	UorB: Unilateral or Bilateral	0.245
BD4: Face recognition burden	BD5: Use of electronic devices burden	0.231
BD1: Mobility burden	BD2: Scene viewing burden	0.217
BD5: Use of electronic devices burden	BD7: Moods and feelings burden	0.215
BD1: Mobility burden	BD3: Activities of daily living burden	0.198
BD2: Scene viewing burden	BD4: Face recognition burden	0.195
BD4: Face recognition burden	BD7: Moods and feelings burden	0.193
UorB: Unilateral or Bilateral	BD5: Use of electronic devices burden	0.191
UorR: Urban or Rural	Trt Dur: Treatment duration	0.190
BD2: Scene viewing burden	BD3: Activities of daily living burden	0.184
Sex	UorB: Unilateral or Bilateral	0.182
BD2: Scene viewing burden	BD6: Hand-eye coordination burden	0.168
Trt Dur: Treatment duration	BD7: Moods and feelings burden	0.146
BD2: Scene viewing burden	BD7: Moods and feelings burden	0.144
BD6: Hand-eye coordination burden	BD7: Moods and feelings burden	0.141
BD1: Mobility burden	BD4: Face recognition burden	0.126
BD4: Face recognition burden	BD6: Hand-eye coordination burden	0.125
BD3: Activities of daily living burden	BD4: Face recognition burden	0.122
Trt Dur: Treatment duration	BD6: Hand-eye coordination burden	0.116
Age	Sex	0.113
BD5: Use of electronic devices burden	BD6: Hand-eye coordination burden	0.108
BD3: Activities of daily living burden	BD5: Use of electronic devices burden	0.108
UorR	Welfare	-0.124
Welfare	Trt Dur: Treatment duration	-0.218

Supplementary Table S6 (a). Validated QOL summary scores according to nAMD laterality.

QOL score	Unilateral	Bilateral	P*
NEI-VFQ-25 score (n)	86	67	**<0.001**
Mean (SD)	75 (15.5)	57.7 (19.2)	
Range (Min; Max)	(31.1; 97.9)	(19.1; 98.0)	
Median (Q1; Q3)	78.6 (67.3; 86.6)	57.5 (43.1; 72.5)	
PHQ-9 summary score (n)	85	65	**0.005**
0–4 None/Minimal	57 (67.1%)	27 (41.5%)	
5–9 Mild	19 (22.3%)	25 (38.5%)	
10–14 Moderate	7 (8.2%)	7 (10.8%)	
15–19 Moderately severe	1 (1.2%)	6 (9.2%)	
20–27 Severe	1 (1.2%)	0 (0%)	
EQ-5D-5L utility (n)	85	64	**<0.001**
Mean (SD)	0.9 (0.1)	0.8 (0.2)	
Range (Min; Max)	(0.4; 1)	(0.3; 1)	
Median (Q1; Q3)	0.9 (0.9; 1)	0.8 (0.7; 0.9)	

Abbreviations: EQ-5D-5L, EuroQol 5-Dimension 5-Level Questionnaire; Min, minimum; Max, maximum; nAMD, neovascular age-related macular degeneration; NEI-VFQ-25, National Eye Institute 25-Item Visual Function Questionnaire; PHQ-9, Patient Health Questionnaire-9; Q, quartile; QOL, quality of life; SD, standard deviation.
*P following the Chi-square or the Fisher's exact test (the latter one if more than 20% of subcategories have frequencies lower than 5) in categorical variables and unpaired Mann-Whitney-Wilcoxon test in continuous variables.

Supplementary Table S6 (b). Validated QOL summary scores according to nAMD severity

QOL score	Severe	Non-severe	P*
NEI-VFQ-25 score (n)	79	71	**<0.001**
Mean (SD)	60.5 (20.7)	75.0 (14.5)	
Range (Min; Max)	(19.1; 97.1)	(37.9; 97.9)	
Median (Q1; Q3)	60.2 (43.1; 77.3)	78.5 (64.4; 87.2)	
PHQ-9 summary score (n)	78	69	**<0.001**
0–4 None/Minimal	34 (43.6%)	48 (69.6%)	
5–9 Mild	26 (33.3%)	17 (24.6%)	
10–14 Moderate	11 (14.1%)	3 (4.3%)	
15–19 Moderately severe	7 (9.0%)	0 (0%)	
20–27 Severe	0 (0%)	1 (1.5%)	
EQ-5D-5L utility (n)	78	68	**<0.001**
Mean (SD)	0.8 (0.2)	0.9 (0.1)	
Range (Min; Max)	(0.3; 1)	(0.4; 1)	
Median (Q1; Q3)	0.8 (0.7; 1)	0.9 (0.8; 1)	

Abbreviations: EQ-5D-5L, EuroQol 5-Dimension 5-Level Questionnaire; Min, minimum; Max, maximum; nAMD, neovascular age-related macular degeneration; NEI-VFQ-25, National Eye Institute 25-Item Visual Function Questionnaire; PHQ-9, Patient Health Questionnaire-9; Q, quartile; QOL, quality of life; SD, standard deviation.
*P following Chi-square or Fisher's exact test (if more than 20% of subcategories have frequencies lower than 5) in categorical variables and unpaired Mann-Whitney-Wilcoxon test in continuous variables.

Supplementary Table S7 (a). NEI-VFQ-25 lower domains for the overall population.

QOL score	All patients
General health (n)	152
Mean (SD)	47.0 (18.8)
Range (Min; Max)	(0; 100)
Median (Q1; Q3)	50 (25; 50)
General vision (n)	151
Mean (SD)	58.5 (21.4)
Range (Min; Max)	(0; 80)
Median (Q1; Q3)	60 (40; 80)
Ocular pain (n)	153
Mean (SD)	78.2 (19.2)
Range (Min; Max)	(25; 100)
Median (Q1; Q3)	87.5 (62.5; 100)
Near activities (n)	152
Mean (SD)	56.1 (23.7)
Range (Min; Max)	(0; 100)
Median (Q1; Q3)	58.3 (41.7; 75)
Distance activities (n)	152
Mean (SD)	66.0 (18.7)
Range (Min; Max)	(16.7; 100)
Median (Q1; Q3)	66.7 (56.2; 75)
Social functioning (n)	150
Mean (SD)	74.7 (20.2)
Range (Min; Max)	(12.5; 100)
Median (Q1; Q3)	75 (62.5; 87.5)
Mental health (n)	153
Mean (SD)	64.9 (25.9)
Range (Min; Max)	(0; 100)
Median (Q1; Q3)	68.7 (50; 87.5)
Role difficulties (n)	152
Mean (SD)	67.3 (23.9)
Range (Min; Max)	(0; 100)
Median (Q1; Q3)	75 (50; 87.5)
Dependency (n)	151
Mean (SD)	74.8 (24.3)
Range (Min; Max)	(8.3; 100)
Median (Q1; Q3)	75 (58.3; 100)
Driving (n)	92
Mean (SD)	60.3 (30.5)
Range (Min; Max)	(0; 100)
Median (Q1; Q3)	68.7 (37.5; 87.5)
Color vision (n)	148
Mean (SD)	81.9 (19.7)
Range (Min; Max)	(0; 100)
Median (Q1; Q3)	75 (75; 100)
Peripheral vision (n)	146
Mean (SD)	63.4 (23.5)
Range (Min; Max)	(0; 100)
Median (Q1; Q3)	75 (50; 75)

Abbreviations: Min, minimum; Max, maximum; nAMD, neovascular age-related macular degeneration; NEI-VFQ-25, National Eye Institute 25-Item Visual Function Questionnaire; Q, quartile; QOL, quality of life; SD, standard deviation.

Supplementary Table S7 (b). NEI-VFQ-25 lower domains according to nAMD laterality.

QOL score	Unilateral	Bilateral	P*
General health (n)	85	67	0.175
Mean (SD)	49.1 (16.6)	44.4 (21.2)	
Range (Min; Max)	(0; 100)	(0; 75)	
Median (Q1; Q3)	50 (50; 50)	50 (25; 50)	
General vision (n)	85	66	**<0.001**
Mean (SD)	65.9 (17.7)	49.1 (22.2)	
Range (Min; Max)	(20; 80)	(0; 80)	
Median (Q1; Q3)	80 (60; 80)	60 (40; 60)	
Ocular pain (n)	86	67	**0.001**
Mean (SD)	83.0 (16.3)	72.0 (21.0)	
Range (Min; Max)	(37.5; 100)	(25; 100)	
Median (Q1; Q3)	87.5 (75; 100)	75 (56.2; 87.5)	
Near activities (n)	86	66	**<0.001**
Mean (SD)	65.0 (20.9)	44.5 (22.2)	
Range (Min; Max)	(0; 100)	(0; 91.7)	
Median (Q1; Q3)	66.7 (50; 75)	50 (25; 58.3)	
Distance activities (n)	85	67	**<0.001**
Mean (SD)	72.2 (15.8)	58.1 (19.1)	
Range (Min; Max)	(25; 100)	(16.7; 100)	
Median (Q1; Q3)	75 (66.7; 83.3)	58.3 (50; 75)	
Social functioning (n)	85	65	**<0.001**
Mean (SD)	81.6 (16.9)	65.6 (20.6)	
Range (Min; Max)	(25; 100)	(12.5; 100)	
Median (Q1; Q3)	87.5 (75; 100)	75 (50; 75)	
Mental health (n)	86	67	**<0.001**
Mean (SD)	72.9 (22.7)	54.6 (26.3)	
Range (Min; Max)	(6.2; 100)	(0; 100)	
Median (Q1; Q3)	75 (62.5; 93.7)	56.2 (34.4; 75)	
Role difficulties (n)	86	66	**<0.001**
Mean (SD)	74.8 (21.6)	57.6 (23.5)	
Range (Min; Max)	(25; 100)	(0; 100)	
Median (Q1; Q3)	75 (62.5; 100)	56.2 (40.6; 75)	
Dependency (n)	85	66	**<0.001**
Mean (SD)	84.0 (18.9)	62.9 (25.6)	
Range (Min; Max)	(25; 100)	(8.3; 100)	
Median (Q1; Q3)	91.7 (75; 100)	62.5 (41.7; 83.3)	
Driving (n)	59	33	**<0.001**
Mean (SD)	70.1 (23.8)	42.8 (33.7)	
Range (Min; Max)	(0; 100)	(0; 100)	
Median (Q1; Q3)	75 (62.5; 87.5)	37.5 (0; 62.5)	
Color vision (n)	85	63	**0.004**
Mean (SD)	85.9 (17.4)	76.6 (21.5)	
Range (Min; Max)	(0; 100)	(0; 100)	
Median (Q1; Q3)	100 (75; 100)	75 (75; 100)	
Peripheral vision (n)	82	64	**<0.001**
Mean (SD)	69.5 (22.6)	55.5 (22.5)	
Range (Min; Max)	(25; 100)	(0; 100)	
Median (Q1; Q3)	75 (50; 75)	50 (50; 75)	

Abbreviations: Min, minimum; Max, maximum; nAMD, neovascular age-related macular degeneration; NEI-VFQ-25, National Eye Institute 25-Item Visual Function Questionnaire; Q, quartile; QOL, quality of life; SD, standard deviation.
*P following unpaired Mann-Whitney-Wilcoxon test.

Supplementary Table S7 (c). NEI-VFQ-25 lower domains according to nAMD severity.

QOL score	Severe	Non-severe	P*
General health (n)	79	71	0.051
Mean (SD)	44.0 (20.9)	50.3 (16.1)	
Range (Min; Max)	(0; 100)	(25; 100)	
Median (Q1; Q3)	50 (25; 50)	50 (50; 50)	
General vision (n)	79	70	**<0.001**
Mean (SD)	50.6 (22.6)	67.1 (16.3)	
Range (Min; Max)	(0; 80)	(20; 80)	
Median (Q1; Q3)	60 (40; 60)	80 (60; 80)	
Ocular pain (n)	79	71	**0.046**
Mean (SD)	75.2 (20.3)	81.7 (17.8)	
Range (Min; Max)	(25; 100)	(25; 100)	
Median (Q1; Q3)	75 (62.5; 87.5)	87.5 (75; 100)	
Near activities (n)	78	71	**<0.001**
Mean (SD)	48.8 (24.4)	63.7 (20.5)	
Range (Min; Max)	(0; 100)	(0; 100)	
Median (Q1; Q3)	50 (25; 66.7)	66.7 (50; 75)	
Distance activities (n)	78	71	**<0.001**
Mean (SD)	60.0 (19.4)	72.1 (15.9)	
Range (Min; Max)	(16.7; 100)	(16.7; 100)	
Median (Q1; Q3)	58.3 (50; 75)	75 (62.5; 83.3)	
Social functioning (n)	76	71	**<0.001**
Mean (SD)	67.6 (22.3)	81.9 (14.8)	
Range (Min; Max)	(12.5; 100)	(37.5; 100)	
Median (Q1; Q3)	75 (50; 87.5)	87.5 (75; 100)	
Mental health (n)	79	71	**<0.001**
Mean (SD)	56.6 (26.3)	73.7 (22.9)	
Range (Min; Max)	(0; 100)	(6.2; 100)	
Median (Q1; Q3)	56.2 (34.4; 81.2)	75 (62.5; 93.7)	
Role difficulties (n)	79	70	**0.002**
Mean (SD)	61.5 (25.3)	74.3 (20.8)	
Range (Min; Max)	(0; 100)	(25; 100)	
Median (Q1; Q3)	62.5 (50; 75)	75 (62.5; 87.5)	
Dependency (n)	78	70	**<0.001**
Mean (SD)	66.3 (26.7)	84.3 (17.8)	
Range (Min; Max)	(8.3; 100)	(33.3; 100)	
Median (Q1; Q3)	75 (41.7; 91.7)	91.7 (75; 100)	
Driving (n)	38	53	0.076
Mean (SD)	52.0 (34.8)	66.3 (26.1)	
Range (Min; Max)	(0; 100)	(0; 100)	
Median (Q1; Q3)	62.5 (25; 75)	75 (62.5; 87.5)	
Color vision (n)	74	71	**0.001**
Mean (SD)	76.7 (22.0)	87.3 (15.7)	
Range (Min; Max)	(0; 100)	(25; 100)	
Median (Q1; Q3)	75 (75; 100)	100 (75; 100)	
Peripheral vision (n)	74	69	**0.004**
Mean (SD)	57.8 (24.1)	69.9 (21.7)	
Range (Min; Max)	(0; 100)	(25; 100)	
Median (Q1; Q3)	50 (50; 75)	75 (50; 75)	

Abbreviations: Min, minimum; Max, maximum; nAMD, neovascular age-related macular degeneration; NEI-VFQ-25, National Eye Institute 25-Item Visual Function Questionnaire; Q, quartile; QOL, quality of life; SD, standard deviation.
*P following unpaired Mann-Whitney-Wilcoxon test.

Supplementary Table S8. Pearson correlation between the scores obtained in the subscales of the NEI-VFQ-25 and in the burden domains of the newly developed questionnaire, overall study population

		Burden domains						
		Mobility burden	Scene viewing burden	Activities of daily living burden	Face recognition burden	Use of electronic devices burden	Hand eye coordination burden	Moods and feelings burden
**NEI-VFQ-25 subscales***	General Health	0.313	0.370	0.365	0.343	0.374	0.475	0.403
	General Vision	0.601	0.601	0.594	0.644	0.641	0.703	0.578
	Ocular Pain	0.469	0.407	0.416	0.384	0.414	0.423	0.441
	Near Activities	0.608	0.645	0.684	0.691	0.642	0.668	0.627
	Distance Activities	0.686	0.654	0.673	0.735	0.737	0.706	0.680
	Social Functioning	0.657	0.621	0.587	0.661	0.660	0.599	0.561
	Mental Health	0.713	0.708	0.692	0.749	0.742	0.691	0.729
	Role Difficulties	0.630	0.588	0.582	0.646	0.635	0.588	0.566
	Dependency	0.677	0.678	0.606	0.684	0.701	0.644	0.634
	Driving	0.568	0.614	0.559	0.571	0.575	0.573	0.532
	Color Vision	0.528	0.471	0.468	0.548	0.532	0.471	0.497
	Peripheral Vision	0.573	0.538	0.523	0.600	0.532	0.538	0.480

Pearson's correlation coefficient. In order to harmonize the two scales, the original scores of the NEI-VFQ-25 subscales were transformed following the formula x→100-x, so that it follows the rule "the higher score, the greater difficulty for a patient", which is in line with the scoring used for the burden domains questionnaire.

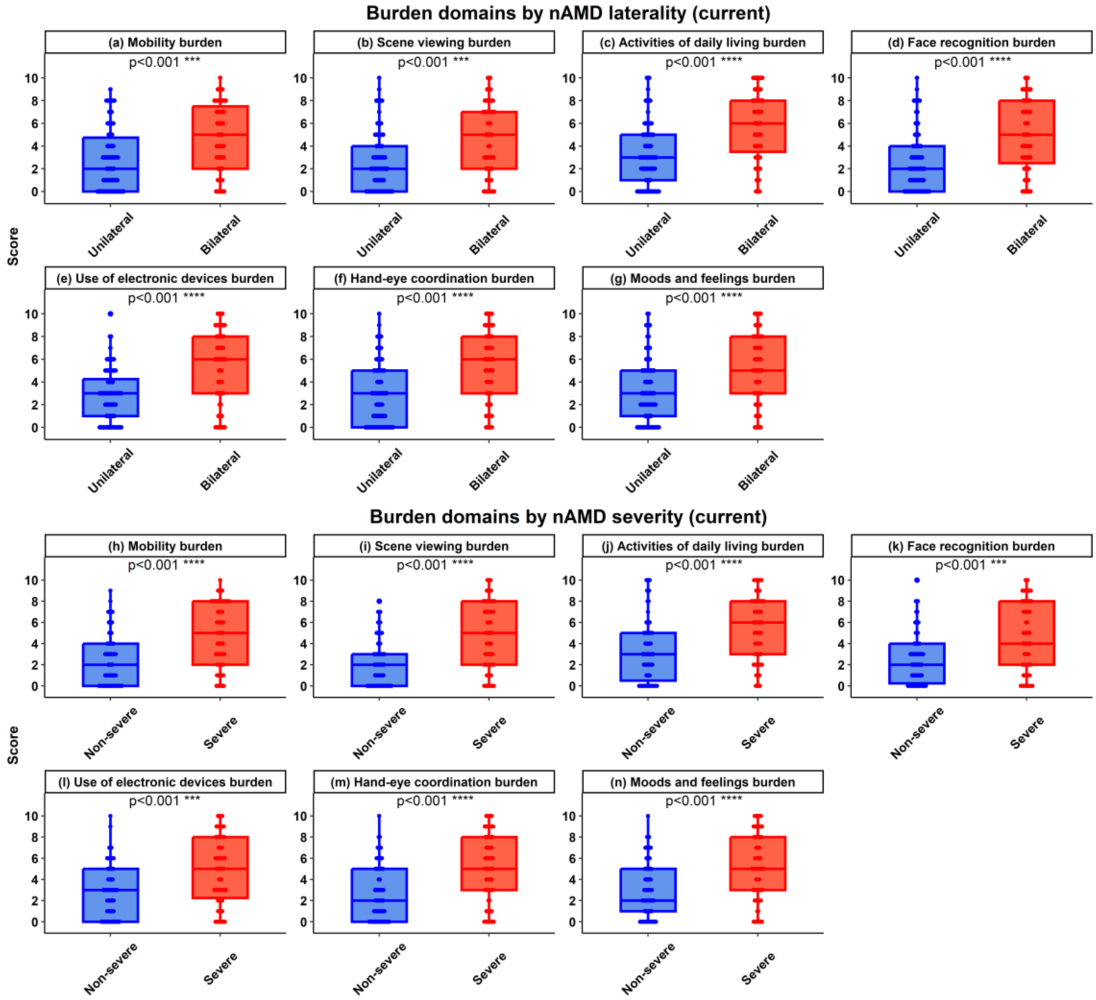

Supplementary Figure S1. nAMD daily life burden domain scores according to subgroups.

The daily life burden scores were significantly higher in all 7 domains in the bilateral subgroup compared with the unilateral subgroup (a-g), and in the severe subgroup compared with the non-severe subgroup (h-n). Mann-Whitney test. ***P<0.001; ****P<0.0001.

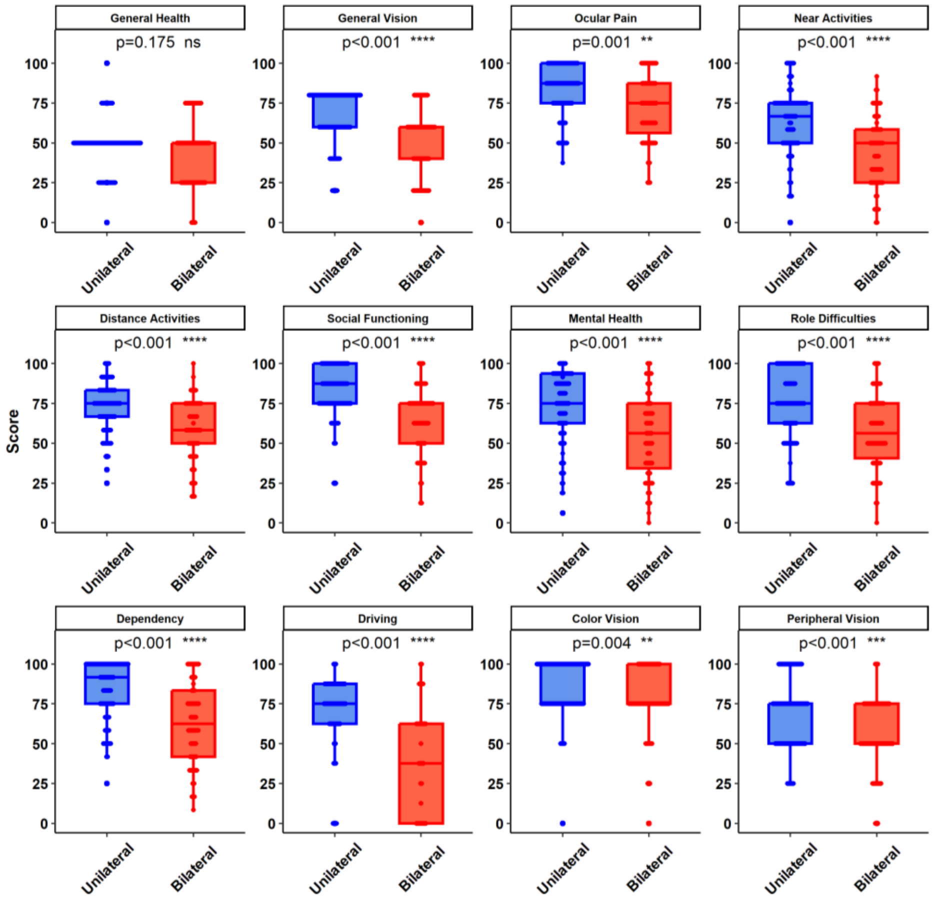

Supplementary Figure S2. NEI-VFQ-25 lower domains according to nAMD laterality.

Abbreviations: nAMD, neovascular age-related macular degeneration; NEI-VFQ-25, National Eye Institute 25-Item Visual Function Questionnaire; ns, not significant.
**Statistically significant result at the level of 0.01; ***Statistically significant result at the level of 0.001; ****Statistically significant result at the level of 0.0001.
These Box plots show the 5-number summary of a dataset: the minimum score (the end of the bottom whisker), first (lower) quartile (the lower line of the box), median (the line that divides the box into two parts), third (upper) quartile (the upper line of the box), and the maximum score (the end of the top whisker).

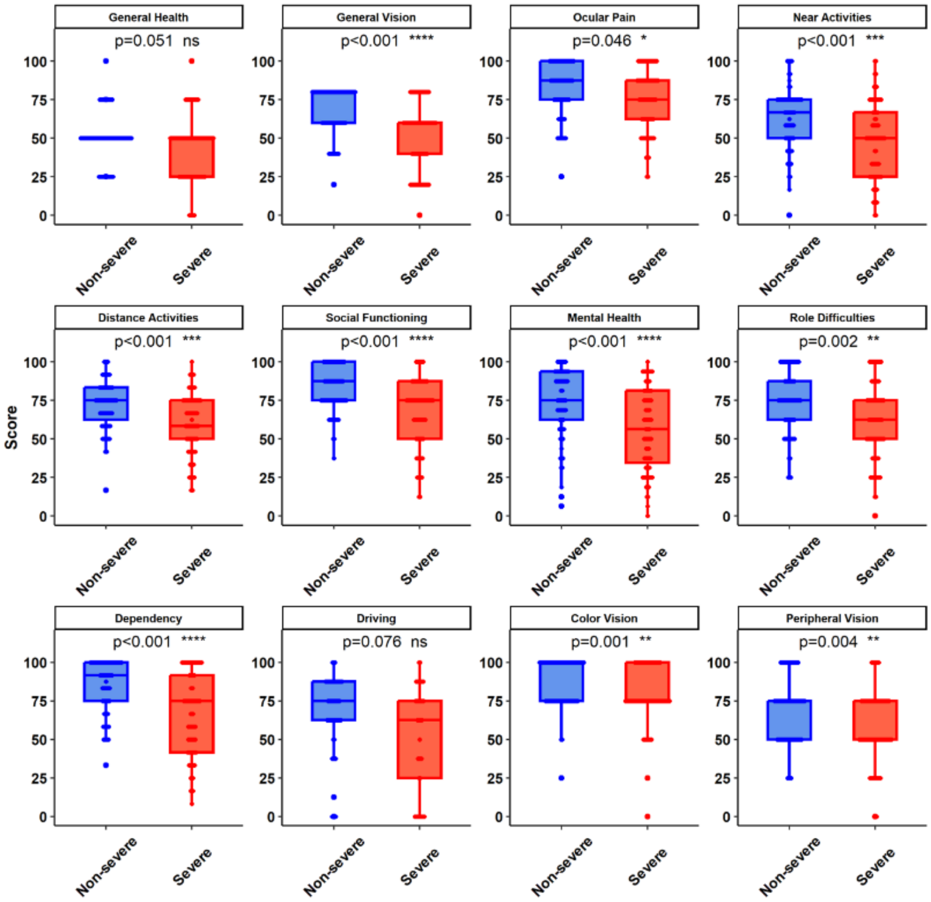

Supplementary Figure S3. NEI-VFQ-25 lower domains according to nAMD severity.

Abbreviations: nAMD, neovascular age-related macular degeneration; NEI-VFQ-25, National Eye Institute 25-Item Visual Function Questionnaire; ns, not significant.
*Statistically significant result at the level of 0.05; **Statistically significant result at the level of 0.01; ***Statistically significant result at the level of 0.001; ****Statistically significant result at the level of 0.0001.
These Box plots show the 5-number summary of a dataset: the minimum score (the end of the bottom whisker), first (lower) quartile (the lower line of the box), median (the line that divides the box into two parts), third (upper) quartile (the upper line of the box), and the maximum score (the end of the top whisker).

Supplementary information: Newly developed Questionnaire in the current study.

Contents

1. Introduction 17

2. Burden and impediments on daily life due to age-related macular disease.....19

Introduction

Neovascular age-related macular degeneration (nAMD) is a disease where the retina, the part of the eye that plays an important role in seeing things, develops abnormalities that make it difficult to see. The main symptoms are loss, distortion, and dimness of vision in the centre of the field of vision.

The following questions are designed to ask you about the problems and inconvenience you experience in your daily life due to **nAMD (your disease)**. Please read the questions and circle the answer that best describes your condition.

These questions are designed to give you a better idea of how **nAMD (your disease)** may affect your everyday life. There are no right or wrong answers, so please read the questions thoroughly and answer them based on your own thoughts.

Notes

1. Please answer the questions yourself.
2. Please answer all the questions.
3. Please circle the answers that apply to you.
4. If you are not sure which answer to give, please choose the answer that best describes your answer.
5. **Eye diseases other than nAMD (mainly cataracts: blurred vision), diseases other than eye diseases (joint pain, difficulty in hearing, pollakiuria, stiffness or numbness of hands or feet, etc.), and burdens or problems due to your age (presbyopia, memory loss, etc.). Please answer the questions excluding these diseases as much as possible.**

Burden and hindrance to daily life due to age-related macular degeneration

For each of the following questions, **(1) when you first heard of age-related macular degeneration (if you first heard of age-related macular degeneration in one eye and then both eyes, please answer regarding the cases of one eye.), and (2) now (the present time)**, please answer the problems or inconveniences you have in your daily life as a result of age-related macular degeneration.

For (1), please answer by recalling as much as possible the time when you were first told by your doctor that you had age-related macular degeneration.

Please answer the question by rating the degree of difficulty or inconvenience on a scale from **0** (no difficulty or inconvenience at all) to **10** (much difficulty or inconvenience and unable to do anything). If **one of the examples applies to you,** please answer about it. If more than one example applies to you, please answer the one that is the most troubling.

<u>If you wear glasses or contacts, please describe when you wear them. Please answer all questions as if you use them, even if you only use them occasionally. Also, please answer all questions in the state of seeing with both eyes.</u>

Questions are on the next page.

Sample

(1) When age-related macular degeneration occurs

If one of the examples applies to you, please circle **the number** that best describes it. If multiple examples apply to you, please answer about the one that is troubling you most.

0 1 2 3 4 5 6 7 8 9 10

No difficulty or inconvenience at all

Much difficulty or inconvenience and unable to do anything

Burden and impediments on daily life due to age-related macular disease

For each of the following questions, please describe any problems or inconvenience you have in your daily life due to having nAMD **(1) when you first heard that you had nAMD (if you have developed nAMD from monocular to binocular, please describe when you first had your monocular nAMD)**, and **(2) now (at present)**, **compared to before you became ill**. In relation to (1), please try to remember the first time you were diagnosed with nAMD from your doctor.

Please rate your level of difficulty or inconvenience on a scale from "**0**: no difficulty or inconvenience at all" to "**10**: much difficulty or inconvenience and unable to do anything". **If one of the examples applies to you**, please answer about it. If multiple examples apply to you, please answer about the one that has troubled you most. **<u>If you use eyeglasses or contact lenses, please tell us about the circumstances when you use them. Please answer all questions as if you were using them, even if you only use them occasionally.</u>**

1. [Mobility]

Examples：

- Complex driving
  - Pulling into a parking lot
  - Reversing a car
  - Overtaking a car in front
  - Turning onto a street
  - Entering an indoor parking lot such as a garage from outside
- Difficulties in walking
  - Going down the stairs (especially the last step)
  - Bumping into objects or people/unable to avoid them
  - Walking up or down the hill or uneven surfaces
  - Entering a dark room from a brightly lit outdoor space
  - Falling easily
- Assessing the distance correctly (in a situation such as playing golf, etc.)
- Judging the depth and perspective correctly

Does your nAMD cause you any problems or inconvenience in your daily life in terms of 'mobility'?

① At the onset of nAMD

If one of the examples applies to you, please circle **the number** that best describes it. If multiple examples apply to you, please answer about the one that has troubled you most.

"No difficulty or inconvenience at all" 0 1 2 3 4 5 6 7 8 9 10 "Much difficulty or inconvenience and unable to do anything"

② Now (at present)

If one of the examples applies to you, please circle **the number** that best describes it. If multiple examples apply to you, please answer about the one that is troubling you most.

"No difficulty or inconvenience at all" 0 1 2 3 4 5 6 7 8 9 10 "Much difficulty or inconvenience and unable to do anything"

2. [Scene viewing]

Examples：

- Identifying similarly coloured and shaped objects such as shampoo and conditioner, salt and pepper, green vegetables (Japanese mustard spinach, spinach, bok choy, etc.)
- Identifying whether lines on a piece of paper/graph or manuscript paper are straight or crooked

Does your nAMD cause you any problems or inconvenience in your daily life in terms of 'scene viewing'?

① At the onset of nAMD

If one of the examples applies to you, please circle **the number** that best describes it. If multiple examples apply to you, please answer about the one that has troubled you most.

"No difficulty or inconvenience at all" 0 1 2 3 4 5 6 7 8 9 10 "Much difficulty or inconvenience and unable to do anything"

② Now (at present)

If one of the examples applies to you, please circle **the number** that best describes it. If multiple examples apply to you, please answer about the one that is troubling you most.

"No difficulty or inconvenience at all" 0 1 2 3 4 5 6 7 8 9 10 "Much difficulty or inconvenience and unable to do anything"

3. [Activities related to daily life]

Examples：

- Reading through a novel or book completely
- Reading a long article in a newspaper
- Watching sports or drama on TV for a long time
- Applying makeup (especially around the eyes and other fine areas)
- Putting toothpaste on your toothbrush
- Coordinating your clothes (colours, designs, etc)
- Doing detailed work such as sewing, carpentry or repairing machinery, etc.
- Distinguishing between socks of similar colours
- Noticing stains on your clothes
- Getting tired eyes easily

Does your nAMD cause you any problems or inconvenience in your daily life in terms of 'activities related to daily life'?

① At the onset of nAMD

If one of the examples applies to you, please circle **the number** that best describes it. If multiple examples apply to you, please answer about the one that has troubled you most.

"No difficulty or inconvenience at all" 0 1 2 3 4 5 6 7 8 9 10 "Much difficulty or inconvenience and unable to do anything"

② Now (at present)

If one of the examples applies to you, please circle **the number** that best describes it. If multiple examples apply to you, please answer about the one that is troubling you most.

"No difficulty or inconvenience at all" 0 1 2 3 4 5 6 7 8 9 10 "Much difficulty or inconvenience and unable to do anything"

4. [Recognition of people's expressions or faces]

Examples:

- Recognising a person's reaction or facial expressions even in dark places
- Recognising the faces of acquaintances in the distance
- Telling people's reactions and emotions by their facial expressions
- Accurately recognising people's faces and expressions on the TV screen
- Accurately recognising your face in the mirror

Does your nAMD cause you any problems or inconvenience in your daily life in terms of 'recognition of people's expressions and faces'?

① At the onset of nAMD

If one of the examples applies to you, please circle **the number** that best describes it. If multiple examples apply to you, please answer about the one that has troubled you most.

"No difficulty or inconvenience at all" 0 1 2 3 4 5 6 7 8 9 10 "Much difficulty or inconvenience and unable to do anything"

② Now (at present)

If one of the examples applies to you, please circle **the number** that best describes it. If multiple examples apply to you, please answer about the one that is troubling you most.

"No difficulty or inconvenience at all" 0 1 2 3 4 5 6 7 8 9 10 "Much difficulty or inconvenience and unable to do anything"

5. [Use of electronic devices]

Examples：

- Reading the contents of computer or cell phone screens
- Reading the text on the screen of a bank ATM
- Reading SMS text or emails on a cell/smart phone screen

Does your nAMD cause you any problems or inconvenience in your daily life in terms of 'use of electronic devices'?

① At the onset of nAMD

If one of the examples applies to you, please circle **the number** that best describes it. If multiple examples apply to you, please answer about the one that has troubled you most.

"No difficulty or inconvenience at all" 0 1 2 3 4 5 6 7 8 9 10 "Much difficulty or inconvenience and unable to do anything"

② Now (at present)

If one of the examples applies to you, please circle **the number** that best describes it. If multiple examples apply to you, please answer about the one that is troubling you most.

"No difficulty or inconvenience at all" 0 1 2 3 4 5 6 7 8 9 10 "Much difficulty or inconvenience and unable to do anything"

6. [Hand-eye coordination]

Examples：

- Receiving small objects handed to you
- Filling a cup with fluid without spilling
- Catching or hitting a moving ball
- Picking up food accurately with chopsticks
- Writing long letters by hand
- Writing letters and characters within a small frame
- Threading a needle
- Picking up/moving a pawn in Igo or Shogi

Does your nAMD cause you any problems or inconvenience in your daily life in terms of 'hand-eye coordination'?

① At the onset of nAMD

If one of the examples applies to you, please circle **the number** that best describes it. If multiple examples apply to you, please answer about the one that has troubled you most.

"No difficulty or inconvenience at all" 0 1 2 3 4 5 6 7 8 9 10 "Much difficulty or inconvenience and unable to do anything"

② Now (at present)

If one of the examples applies to you, please circle **the number** that best describes it. If multiple examples apply to you, please answer about the one that is troubling you most.

"No difficulty or inconvenience at all" 0 1 2 3 4 5 6 7 8 9 10 "Much difficulty or inconvenience and unable to do anything"

7. [Mental health]

Examples：

- Anxiety about the future
- Loss of motivation
- Feeling low or depressed
- Loss of confidence
- Social withdrawal

- Feeling miserable

Does your nAMD cause you any problems or inconvenience in your daily life in terms of 'mental health'?

① At the onset of nAMD

If one of the examples applies to you, please circle **the number** that best describes it. If multiple examples apply to you, please answer about the one that has troubled you most.

"Never" 0 1 2 3 4 5 6 7 8 9 10 "Most of the time"

② Now (at present)

If one of the examples applies to you, please circle **the number** that best describes it. If multiple examples apply to you, please answer about the one that is troubling you most.

"Never" 0 1 2 3 4 5 6 7 8 9 10 "Most of the time"

8. [Summary]

a. Do you take any measures (including changing the angle of vision and using spectacles, etc.) to reduce the strain on the eyes caused by nAMD?

☐ Yes

☐ No

b. (If answered 'Yes' to the question above) Has the difficulty or inconvenience you felt '**at the onset of nAMD**' stopped bothering you '**now (at present)**' as a result of the measures you have taken?

☐ Yes

☐ No
